# Regulation of CRISPR-Associated Genes by Rv1776c (CasR) in *Mycobacterium tuberculosis*

**DOI:** 10.3390/biom13020400

**Published:** 2023-02-20

**Authors:** Wenping Wei, Xiaofang Jiang, Li Zhang, Yunjun Yan, Jinyong Yan, Li Xu, Chun-Hui Gao, Min Yang

**Affiliations:** 1 Key Laboratory of Molecular Biophysics of the Ministry of Education, College of Life Science and Technology, Huazhong University of Science and Technology, Wuhan 430074, China; 2 State Key Laboratory of Agricultural Microbiology, College of Life Science and Technology, Huazhong Agricultural University, Wuhan 430070, China; 3 State Key Laboratory of Agricultural Microbiology, College of Resources and Environment, Huazhong Agricultural University, Wuhan 430070, China

**Keywords:** *Mycobacterium tuberculosis*, CRISPR-Cas System, CasR, transcriptional regulation

## Abstract

The CRISPR-Cas system is an adaptive immune system for many bacteria and archaea to defend against foreign nucleic acid invasion, and this system is conserved in the genome of *M. tuberculosis* (*Mtb*). Although the CRISPR-Cas system-mediated immune defense mechanism has been revealed in *Mtb*, the regulation of *cas* gene expression is poorly understood. In this study, we identified a transcription factor, CasR (CRISPR-associated protein repressor, encoded by *Rv1776c*), and it could bind to the upstream DNA sequence of the CRISPR-Cas gene cluster and regulate the expression of *cas* genes. EMSA and ChIP assays confirmed that CasR could interact with the upstream sequence of the *csm6* promoter, both in vivo and in vitro. Furthermore, DNA footprinting assay revealed that CasR recognized a 20 bp palindromic sequence motif and negatively regulated the expression of *csm6.* In conclusion, our research elucidates the regulatory effect of CasR on the expression of CRISPR-associated genes in mycobacteria, thus providing insight into gene expression regulation of the CRISPR-Cas system.

## 1. Introduction

Clustered regularly interspaced short palindromic repeats (CRISPR) and CRISPR-associated (Cas) proteins are found in most (~90%) sequenced archaeal genomes and many (~50%) sequenced bacterial genomes [1,2]. In addition to its role as a prokaryotic adaptive immune system in protecting cells from the invasion of foreign mobile genetic elements, including bacteriophages and plasmids, the CRISPR-Cas system also modulates multiple biological processes such as gene regulation and bacterial virulence, especially pathogen stress responses [3,4,5]. However, the regulatory mechanism of the CRISPR-Cas system is far from clear.

Based on different modes of action and the function of Cas proteins, CRISPR-Cas systems are divided into two classes and six types. The Class 1 CRISPR-Cas system, which includes types I, III, and IV, can form multi-subunit complexes around the Cas7 backbone, whereas, in Class 2 (types II, V, and VI), interference is achieved by a single effector protein comprising multiple nuclease domains [1,5]. CRISPR-Cas systems immunity consists of three distinct immune stages: adaptation, RNA maturation, and interference [6]. The adaptation process involves the insertion of short DNA sequences called spacers, which originated from invading bacteriophages, into the CRISPR arrays to preserve the memory of previous infections. CRISPR arrays are transcribed and processed into CRISPR RNAs (crRNAs) that contain individual spacer sequences and are arranged by repeat fragments [7,8]. During the CRISPR interference stage, Cas proteins bind to individual crRNAs to form effector complexes that recognize complementary sequences (known as ‘protospacer’) in foreign nucleic acids. Once a protospacer is recognized, the target DNA or RNA transcript is degraded by a Cas-crRNA ribonucleoprotein complex [9,10,11].

*Mycobacterium tuberculosis* (*Mtb*), the causative agent of tuberculosis, encodes a complete type III-A CRISPR-Cas system in its genome [12]. The type III CRISPR-Cas system is one of the most refined, powerful, and versatile characterized CRISPR interference complexes. This system comprises Csm1/2/3/4/5/6, and Cas6, which are responsible for crRNA processing and maturation, and two adaptation proteins (Cas1 and Cas2) located next to two CRISPR arrays with 24 and 18 repeats, respectively [13,14,15]. The type III CRISPR-Cas system in *M. bovis* is identical to that in *Mtb*, except that the *csm6* gene is truncated, and that spacer leader sequence is lacking [12]. A recent study has reported that the CRISPR-Cas system can effectively reduce plasmid transformation [16]. Research on the reconstruction and characterization of the *Mtb* CRISPR system in *Escherichia coli* has indicated that mycobacterial immunity to foreign nucleic acids is achieved mainly by cA6-activated Csm6 nuclease activity rather than through DNA cleavage by Csm1 [17]. Therefore, the regulation of *csm6* gene expression is crucial for the biological activity of the CRISPR-Cas system in *Mtb*.

The genome of *Staphylococcus epidermidis* also harbors a type III A-CRISPR-Cas system, in which Csm6 is required for defense against the invasion of bacteriophages; Csm6 can degrade RNA transcripts to ensure efficient immunity under the conditions of slow degradation of target DNA [18]. This system provides effective immunity, but it may also lead to the growth arrest of infected cells during the interference stage, thus necessitating ribonuclease inactivation in the case of bacteriophage clearance [19,20]. Therefore, it is vital for bacteria to precisely regulate the expression of CRISPR-associated genes. In some bacterial species, transcription factors such as CPR (cyclic AMP receptor protein), LRP (leucine-responsive regulatory protein), LeuO (regulator of leucine biosynthesis operon), and H-NS (histone-like nucleoid-structuring protein) regulate the expression of the CRISPR-Cas system by binding to CRISPR promoters [21]. In *Mtb*, Rv2837c (CnpB) controls the expression of the CRISPR-Cas system through a nanoRNA with oligoribonuclease (Orn)-like activity [22]. However, there are few reports on transcription factors regulating the CRISPR-Cas system in mycobacteria.

In the present study, we identified a TetR family transcription factor and named it CasR; it regulated the expression of *cas* genes of the CRISPR-Cas system in *Mtb*. In vitro EMSA and in vivo ChIP assays showed that CasR specifically bound to the promoter of *csm6* in the CRISPR-Cas system. The β-galactosidase activity assay and qPCR assay indicated that CasR negatively regulated the expression of *csm6*. We found that the CasR regulator was conserved in multiple mycobacteria, suggesting that it may control the CRISPR-Cas system broadly. Our findings deepen the understanding of gene regulation of the bacterial CRISPR-Cas system.

## 2. Materials and Methods

### 2.1. Bacterial Strains, Plasmids, and Reagents

*Mtb* H_37_Ra (purchased from ATCC) was cultivated in Middlebrook 7H9 medium containing 0.05% glycerol and 10% oleic acid–albumin–dextrose–catalase (OADC) enrichment. *Escherichia coli* BL21 (DE3) was incubated in Luria-Bertani (LB) medium and used for protein expression. The restriction enzymes, T4 DNA Ligase, Taq DNA polymerase, and DNase I were purchased from New England Biolabs (Beverly, MA, USA) and Thermo Fisher Scientific (Waltham, MA, USA). All antibiotics, including kanamycin (Kan) and ampicillin (Amp), were purchased from Sigma-Aldrich (Darmstadt, Germany). Antiserum was obtained from Dia-An Biotechnology Co., Ltd., (Wuhan, China). All constructed plasmids and strains are listed in Table S1.

### 2.2. Plasmid Construction and Protein Purification

To construct the *casR*-overexpressing strain, the *casR* coding sequence was amplified via PCR using a pair of respective primers (Table S2). The PCR products were digested with *Eco*RI/*Xba*I and then cloned into the *Eco*RI/*Xba*I-digested pMV261 vector with L5 regulatory promoter using T4 DNA ligase. The pMV261-*casR* recombinant plasmid was electroporated into *M. tuberculosis* H_37_Ra to generate an overexpression strain. For CasR and other regulator proteins’ purification, the PCR products were inserted into the pET28a vector of *Eco*RI/*Xba*I digestion using recombinant enzymes (Yeasen, Wuhan, China). Then, the recombinant plasmids were transformed into BL21 competent cells, and transformants were selected on plates containing 50 μg/mL Kan. Then, the cultures were grown at 37 °C until the OD_600_ (optical density) reached ~ 0.6 and were induced by adding 0.3 mM isopropyl β-D-thiogalactoside (IPTG, CAS 367-93-1, Amresco, Solon, OH, USA) for 16 h at 16 °C. The recombinant proteins were purified using an Ni^2+^-affinity column, as described previously [23]. The purified proteins were analyzed using SDS-PAGE. The supernatant was filtered with a 0.22 μm membrane and was concentrated by centrifugation at 2000× *g* at 4 °C using an ultrafiltration tube (molecular weight cut-off: 3 kDa, Millipore). Finally, the proteins were dialyzed using dialysis buffer (20 mM Tris-HCl, 100 mM NaCl, 10% glycerin, pH 8.0) for 2 h at 4 °C and stored at −80 °C. The protein concentration was determined using the Coomassie brilliant blue protein kit (Sigma-Aldrich, St. Louis, MO, USA).

### 2.3. Electrophoretic Mobility Shift Assay (EMSA)

The DNA probes used in EMSA were 500 base pairs (bp) long, including 400 bp upstream from the start codon and the first 100 bp of each gene coding region. All DNA probes were obtained via PCR using *Mtb* H_37_Ra genomic DNA as a template. The promoter primers for *Rv0324*, *Rv1255c*, *Rv1473A*, *csm6*, *casR*, and *cas6* are listed in Table S2. DNA–protein interactions were performed as previously described, with some modifications [24]. Briefly, 20 μL of reaction mixtures containing 100 ng DNA probes, 0.5–2 μM regulator proteins and reaction buffer (100 mM Tris-HCl, pH 7.5, 7 mM MgCl_2_, 50 mM NaCl, 10% glycerin) were incubated for 30 min at room temperature. Then, the mixtures were electrophoresed into a 5% native polyacrylamide gel containing 0.5 × TBE buffer and ran at 150 V for 1 h. Images were acquired with a Typhoon Scanner (GE healthcare, Buckinghamshire, UK).

### 2.4. Chromatin Immunoprecipitation (ChIP) Assay

In vivo interactions of the CasR protein with its own promoter and potential target gene promoters were analyzed using the ChIP assay, using a previously reported method with some modifications [24]. In order to obtain a high-affinity CasR antibody, the high-purity CasR protein was sent to Dia-An Biotechnology Co., Ltd. (Wuhan, China) for polyclonal antibody production. The *Mtb* H_37_Ra strain was cultured in 100 mL of 7H9 medium supplemented with 10% OADC until OD_600_ reached 1.0. The bacterial solution was fixed with 1% formaldehyde for 30 min at room temperature, then 0.125 M glycine was added to terminate the reaction. Afterwards, cross-linked cells were harvested, washed with 5 mL of pre-cooled PBS, and resuspended in 1 mL of TBSTT-I (20 mM Tris-HCl, pH 7.5, 150 mM NaCl, 0.1% Tween 20, 0.1% Triton X-100, 1 mM protease inhibitor cocktail). The sample was sonicated at 250 W on ice for 3 min, and the average 0.5–1 kb DNA fragment was selected for further experiments. A 100 μL sample of the supernatant was used as the input fraction, whereas the remaining 800 μL was divided into two parts. These two parts were added with preimmunization serum or antiserum against CasR and incubated under rotation for 3 h at 4 °C. The protein complexes were immunoprecipitated with 50 μL of 50% protein A agarose under rotation for 8 h at 4 °C. The immune complexes were washed 5 times with 1 mL of TBSTT-I, 1 time with TBSTT-II (500 mM NaCl), and 1 time with TBSTT-III (containing 0.5% Tween 20 and 0.5% Triton X-100). Finally, the complexes were dissociated with 50 μL of TE (containing 20 mM Tris-HCl and 10 mM EDTA, 0.5% SDS, pH 7.8) in a 65 °C water bath for 2 h. The DNA samples from the ChIP assay were purified and analyzed using PCR. The primer sequences were listed in Table S2. The PCR amplification was performed as follows: pre-denaturation at 95 °C for 5 min, followed by 25 cycles of 95 °C for 20 s, 60 °C for 20 s, and 72 °C for 30 s. The PCR products were separated using 1.5% agarose gel electrophoresis.

### 2.5. DNase I Footprinting Assay

For the footprinting assay, the 500 bp *casRp* DNA fragment at the positions from −400 to +100 of the *casR* translational start site was amplified via PCR using specific primers labeled with 5′-FAM. A DNase I footprinting assay was performed, as described previously [25]. The amplified DNA products were purified with the OMEGA DNA Purification kit (OMEGA), and then subjected to the binding reaction under the same conditions as EMSA. Briefly, a total of 1.5 μg of DNA probes were co-incubated with CasR protein in reaction buffer for 30 min at 25 °C. Then, 0.0025 U DNase I was added to the mixture for digestion at 37 °C for 100 s, and phenol chloroform was added immediately to terminate the reaction. The samples were centrifuged and then precipitated with ethanol at −80 °C overnight. Finally, the DNA precipitates were resuspended in 10 μL of ddH_2_O, and the fragments were subjected to STR sequencing by Tsingke Biological Technology (Wuhan, China). Electropherograms were analyzed using GENEMAPPER software (version 4.0, Applied Biosystems).

### 2.6. β-Galactosidase Activity Assay

A series of operon-*lacZ* fusion plasmids derived from pMV261 were constructed to perform β-gal activity experiments in the *M. smegmatis* strain [26]. The target and control gene promoter fragments were amplified with PCR using two pairs of primers (Table S2), and then cloned into the pMV261-*lacZ* backbone. Similarly, the regulator *casR* was ligated behind the target promoter and cloned into the pMV261-*lacZ* vector together. To construct the *csm6pm*-*casR*-*lacZ* or *casRpm-casR*-*lacZ* plasmid, which were used as a control for promoter mutations, the mutant promoter *csm6pm* or *casRpm* was inserted upstream of the *casR* gene. In addition, the enhanced promoter *hsp60* was inserted into pMV261-*lacZ* as a positive control, and the empty plasmid served as a negative control. All the recombinant plasmids were transformed into the *M. smegmatis* strain to obtain corresponding reporter strains. All the reported strains were verified by PCR, using the upstream pMV261 sequence of the 5′-end of the DNA fragment as a forward primer and the 3′-end of the DNA fragment as the reverse primer. Then, the PCR products were sent to the TsingKe Company for DNA sequencing. The strains were cultured in 5 mL of 7H9 medium until the mid-log phase. The cells were harvested and washed using cold PBS. β-gal activity was measured as described previously [27].

### 2.7. Real-Time Quantitative Reverse-Transcription PCR (qRT-PCR) Assay

The pMV261 empty and *casR*-overexpressing strains were grown to OD_600_ of 1.0 in 100 mL of 7H9 medium. The extraction of mRNA from the cultures and qRT-PCR analysis was performed as described previously [23]. The cDNA was obtained using the HiScript II Q RT kit (Vazyme, Nanjing, China). The qRT-PCR system consisted of 20 μL of solution containing 10 μL of 2 × SYBR Green qPCR mix, 400 nM specific primers, and 1 μL of cDNA. The reactions were performed in a Bio-Rad CFX instrument under the following program: 95 °C for 1 min and 40 cycles of 95 °C for 15 s, 60 °C for 15 s, and 72 °C for 30 s. The expression level of each gene was normalized with *sigA* as an internal reference. Gene relative expression was determined according to the 2^−ΔΔCt^ method [28].

## 3. Results

### 3.1. Identification of Transcription Factors Regulating CRISPR-Cas System Gene Expressions

**Figure 1:**
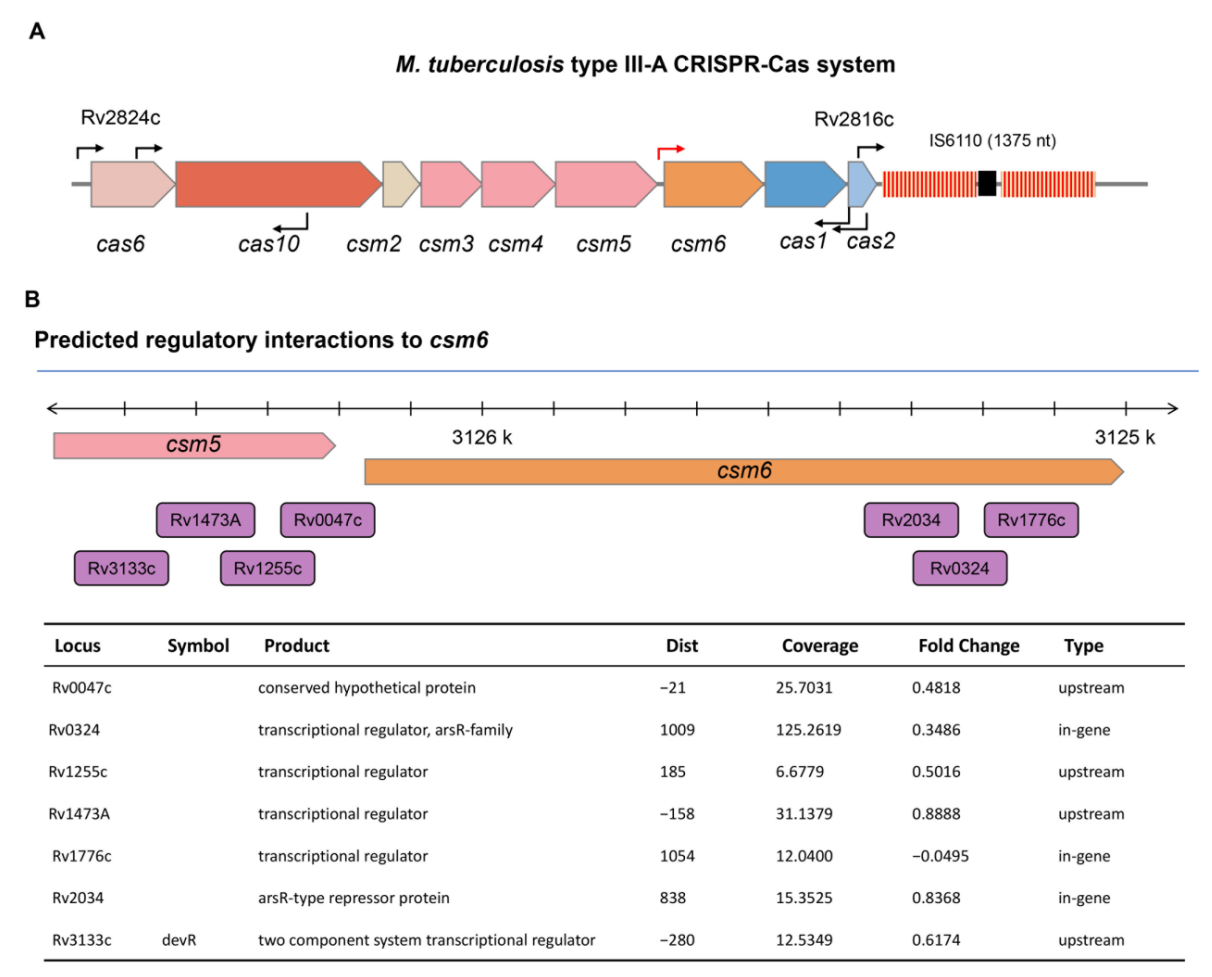
Type III-A CRISPR system of *M. tuberculosis*. (**A**) Genetic organization of the CRISPR-Cas system in *M. tuberculosis*. In *M. tuberculosis*, the CRISPR-Cas system is a type III-A system, and the expression pattern may differ from that of other *cas* genes. An independent transcription start site (TSS) was identified upstream of the gene *csm6* (red arrow). The TSS of other genes are indicated with black arrows in the schematic. (**B**) Predicted transcription factors interacting with Csm6. The blocks in purple refer to the predicted transcription factors, and the locations indicate the maximum coverage of possible binding sites. Fold change is the expression of a target gene (*csm6*) when the corresponding transcription factor is induced, as noted in the TB database. Binding types are either genic or intergenic, based on the location of maximum coverage for the binding site relative to predicted genes.

**Figure 2:**
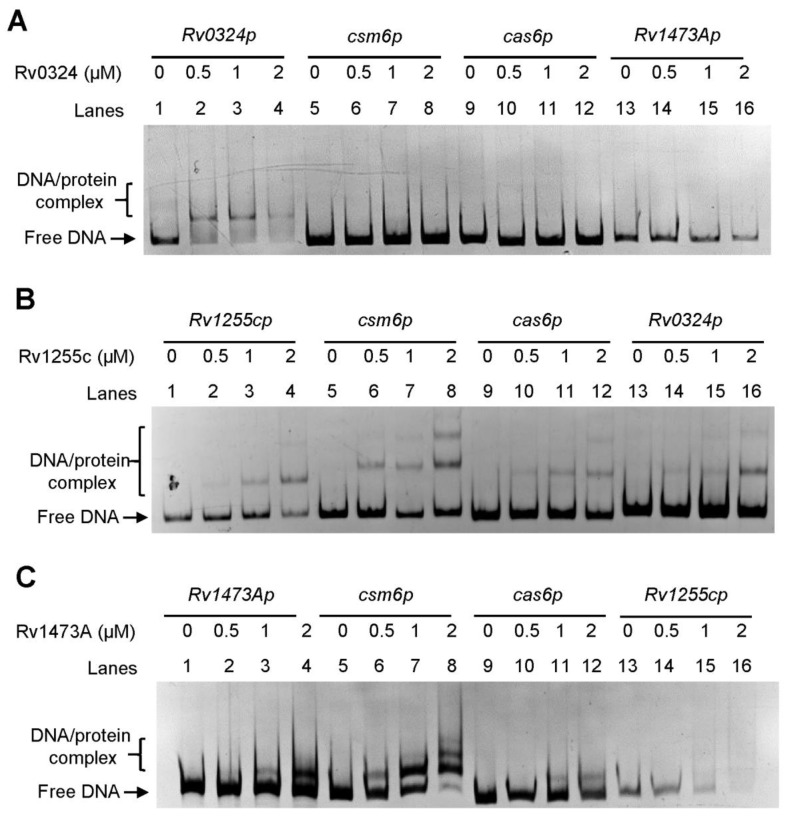
EMSA of binding of transcription factors (**A**) Rv0324, (**B**) Rv1255c, and (**C**) Rv1473A to their own promoters and CRISPR system promoters. The 100 ng target gene *csm6* and 100 ng *cas6* promoters were co-incubated with different concentrations (0–2 μM) of transcription factors. The DNA-protein complex is indicated with square brackets.

In *M. tuberculosis*, the CRISPR-Cas system is a type III-A system. The expression pattern of *csm6* may differ from that of other *cas* genes, and the transcription start site (TSS) has been identified as an independent one upstream of the *csm6* by high-throughput screening (Figure 1A) [29]. To find putative regulators of the CRISPR-Cas system, we queried the TB database [30,31] and retrieved seven regulators encoded by *Rv0047c, Rv0324, Rv1255c, Rv1473A, Rv1776c, Rv2034,* and *Rv3133c* (*devR*), which may interact with the gene promoter region of *csm6* (Figure 1B). Among these regulators, Rv2034 and Rv3133c were both global ones which can regulate multiple biological processes, including hypoxia adaptation, and virulence; moreover, these two transcriptional factors have been extensively investigated [32,33]. However, knowledge of the other transcriptional factors was still lacking. Notably, the ChIP-seq signal of Rv0324 and Rv1473A was 10-fold and 2-fold more intensive than that of Rv2034 and Rv3133c, respectively [31]. In addition, evidence showed that the binding regions of Rv1473A, Rv1255c, and Rv0047c are located upstream of the *csm6* gene, while the binding regions of Rv1776c and Rv0324 are located in the coding region of the *csm6* gene. Based on these findings, we hypothesized that Rv0047c, Rv0324, Rv1255c, Rv1473A, and Rv1776c might be novel regulators of the CRISPR–Cas system in *M. tuberculosis*. To verify our hypothesis, we subsequently cloned, expressed, and purified these regulators. All of these regulators were expressed successfully, except that Rv0047c was completely insoluble and failed to result in purified protein (Figure S1). Furthermore, the EMSA was performed to determine whether the remaining regulators (excluding Rv0047c) could specifically interact with two putative promoters of *csm6* (*csm6p*) and *cas6* (*cas6p*). As shown in Figure 2A, Rv0324 formed a protein–DNA complex shift band with its own promoter, but it did not bind to *csm6p*, *cas6p*, or the promoter region of Rv1473A (*Rv1473Ap*). Moreover, Rv1255c bound to self-promoter, *csm6p*, *cas6p*, *Rv0324p,* and some unrelated promoters, indicating that Rv1255c lacked specific binding activity to DNA (Figure 2B and Figure S2). Similar to Rv1255c, Rv1473A also lacked specific DNA binding activity, except for the different position where its shift band appeared (Figure 2C and Figure S3). After the initial filtration, Rv1776c was screened as the only candidate and named CasR.

### 3.2. CasR Specifically Interacts with csm6 Promoter In Vivo and In Vitro

**Figure 3:**
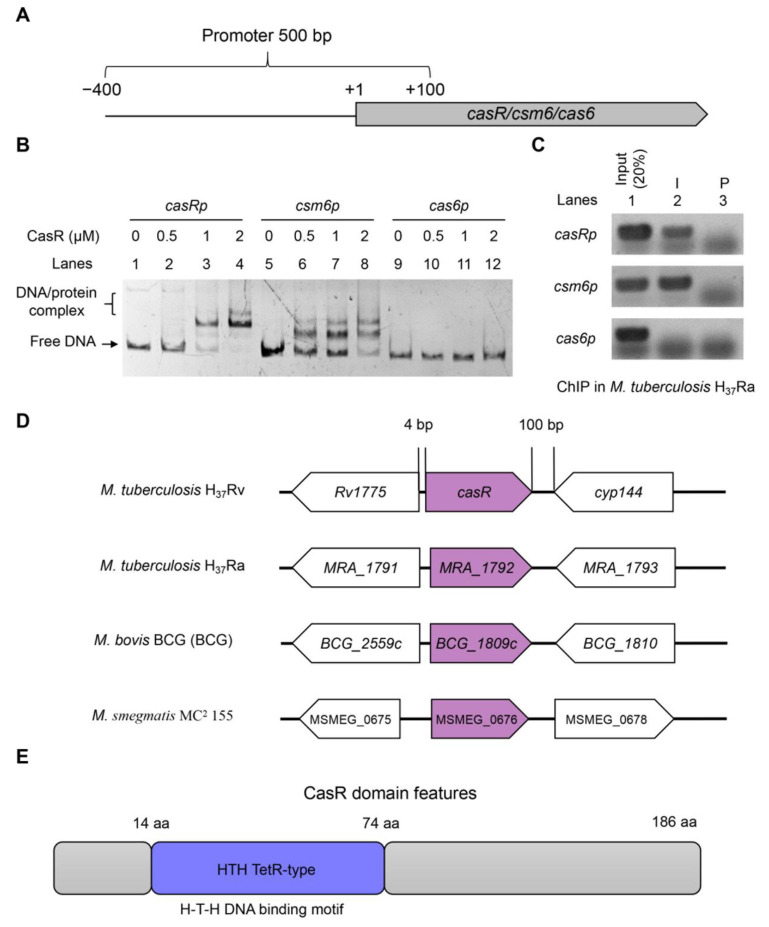
Specific interaction of CasR with the promoter of the *csm6* in CRISPR-Cas system of *M. tuberculosis*. (**A**) Schematic diagram of the promoter fragment used in this experiment. (**B**) EMSA of CasR binding to its own promoter and CRISPR system promoters. The 100 ng *casR* promoter (*casRp*, Lanes 1–4) and 100 ng target gene *csm6* promoter (*csm6p*, Lanes 5–8) were incubated with different concentrations (0–2 μM) of CasR protein. The non-specific promoter *cas6p* (Lanes 9–12) served as a negative control. The DNA–protein complex is indicated with square brackets. (**C**) ChIP assay in the wild-type *M. tuberculosis* H_37_Ra strain. In ChIP, P indicates pre-immune serum, and I denotes antiserum against CasR. The mycobacterial promoter *cas6p* was used as a negative control. (**D**) CasR amino acid sequence with 100% identity in *M. tuberculosis* H_37_Rv, *M. tuberculosis* H_37_Ra, and *M. bovis* BCG. (**E**) Domain characteristics of CasR. CasR contained an HTH TetR_type superfamily domain and a C-terminal domain with unknown function.

We then analyzed the interaction of CasR protein with several DNA probes, whose sizes and characteristics are given in Figure 3A. Notably, when either *casRp* or *csm6p* DNA probes were co-incubated with increasing concentrations of CasR (0, 0.5, 1, and 2 μM), clearly shift bands were observed (Figure 3B, Lanes 1–8). In contrast, CasR did not bind to *cas6p* or the control promoter *groEL1p* (Figure 3B, Lanes 13–16 and Figure S4). These results suggested that CasR could specifically bind to its own promoter region and the promoter of *csm6* in vitro.

A ChIP assay was performed to further confirm the binding of CasR to *casRp* and *csm6p* DNA fragments in *M. tuberculosis*. As shown in Figure 3B, after immunoprecipitation with the CasR antiserum, two DNA fragments were specifically recovered, but they were not recovered after immunoprecipitation with the pre-immune serum (Figure 3C). In contrast, the promoter of *cas6* (as negative control) was not recovered by either CasR antiserum or pre-immune serum (Figure 3C). These results showed that CasR specifically interacted with the upstream DNA sequence of *csm6*, both in vitro and in vivo.

Orthologs of CasR were identified based on sequence similarity and the conservation of adjacent genes, and CasR and its orthologs were annotated as hypothetical proteins (Figure 3D). The CasR region was highly conserved in *M. tuberculosis* H_37_Rv, *M. tuberculosis* H_37_Ra, and *M. bovis* BCG with amino acid identity of 100% but not highly conserved in *M. smegmatis* MC^2^ 155, with an amino acid identity of only 59.55% (Figure S5). The NCBI database showed that the gene *casR* was 561 bp long, and the protein encoded 186 amino acids containing a typical TetR_N superfamily domain with a helix-turn-helix DNA binding motif, and a molecular weight of 20314 Daltons (Figure 3E), suggesting that CasR belongs to the TetR family transcription factors.

### 3.3. CasR Recognizes A 20 bp Palindrome Sequence Motif

**Figure 4:**
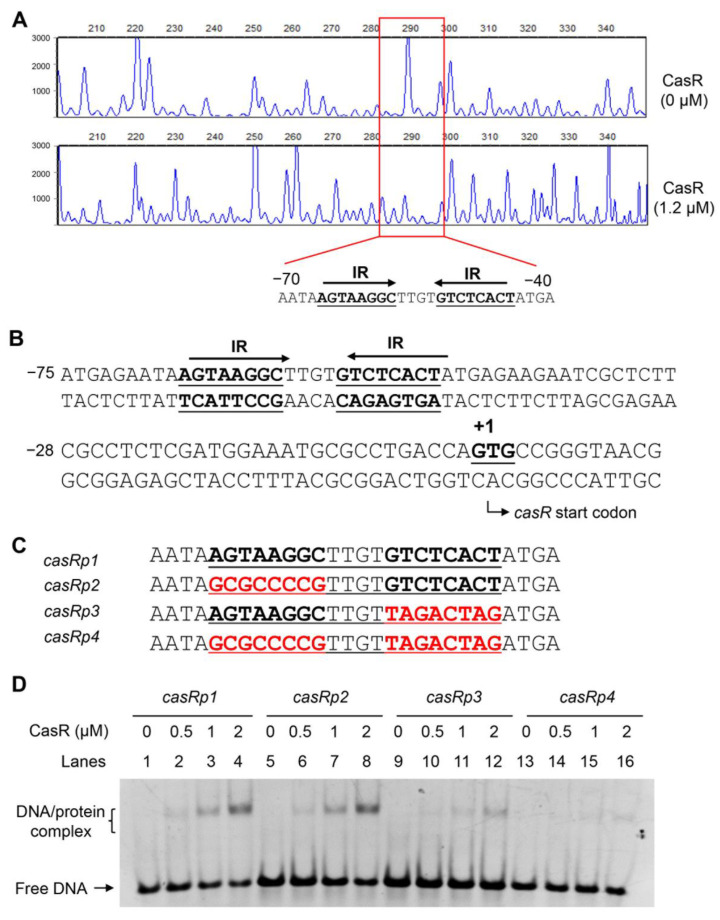
Profile of motif sequence recognized by CasR. (**A**) DNase I footprinting assay. The protective effect of CasR on the promoter DNA against DNase I digestion increased with increasing concentrations of CasR (0 μM, 1.2 μM). The sequences in the protected regions on the coding strand are underlined in the figures. (**B**) Sequence and structural characteristics of the promoter region protected by CasR. The regions protected by CasR are indicated with underlines, and the box indicates the 20 bp sequences containing two inverted repeats (IRs) with a 4 bp interval. The translation start codon of CasR is indicated in bold. (**C**) EMSA of DNA sequences with different mutations. Three mutant DNA probes contain left part mutation, right part mutation, and complete mutation of palindromic structure. (**D**) EMSA of four mutant DNA probes, with or without the IR sequence. Each DNA probe was co-incubated with 0–2 μM CasR protein.

To further identify potential motifs of DNA fragments recognized by CasR, a DNase I footprinting assay was performed. CasR protein was co-incubated with the DNA probe *casRp*, and then digested with DNase I. The results showed that the sequence AATAAGTAAGGCTTGTGTCTCACTATGA, located in the region −70 to −40 on the coding strand, was most significantly protected. In this sequence, a palindromic sequence motif was found to be separated by two partially matched inverted repeats (Figure 4A,B), suggesting that CasR recognized a palindrome sequence motif. Further EMSAs confirmed that a 20 bp motif, AGTAAGGCTTGTGTCTCACT of this sequence (AATAAGTAAGGCTTGTGTCTCACTATGA), was required for recognition by CasR. Four 28 bp DNA probe fragments, designated as *casRp1*–*casRp4*, where *p1* is native DNA and *p2–4* are mutants, were synthesized and subjected to EMSAs (Figure 4C). As shown in Figure 4D, CasR did not bind to the *casRp4* fragments with two mutated inverted repeats; however, it bound to *casRp2* and *casRp3* probes with one intact inverted repeat, although the binding was slightly weaker than that to *casRp1*. These data indicate that half of the palindromic sequence is sufficient for CasR binding. Taken together, our findings indicate that CasR recognized a 20 bp palindrome sequence motif located in the upstream regulatory region of the *casR* promoter.

### 3.4. CasR Binds to csm6 Promoter in the Same Manner as Its Binding to Self-Promoter

**Figure 5:**
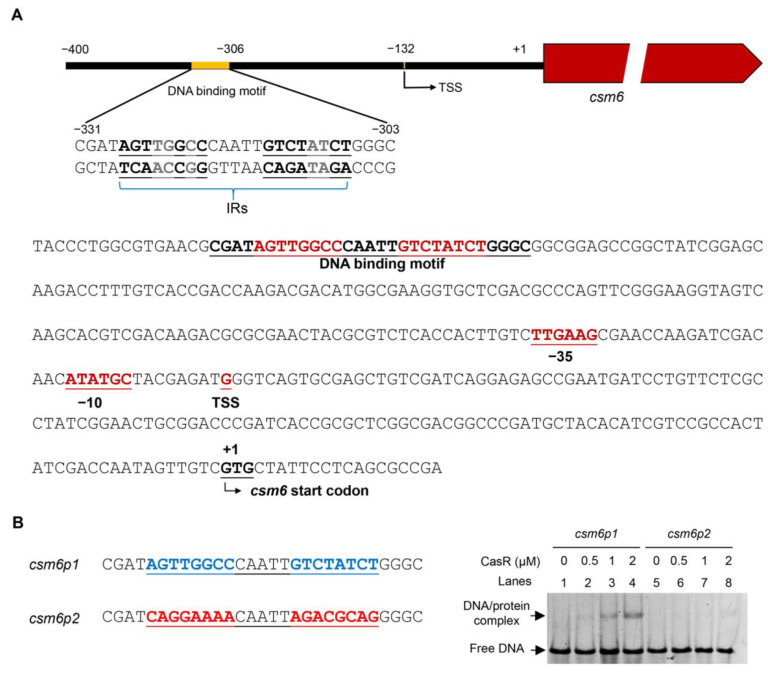
Motif of CasR-binding *csm6* promoter. (**A**) Sequence and structural characterization of CasR-protected *csm6* promoter region. The CasR-protected regions from −331 to −306 are indicated with underlines. Putative −10 and −35 sequences are marked. The approximate transcription start site at position −132 is marked with TSS. (**B**) EMSA of DNA-binding activity of CasR on the DNA probes, with (*csm6p1*, Lanes 1–4) or without IR sequence (*csm6p2*, Lanes 5–8). Either DNA probe was co-incubated with 0–2 μM CasR protein.

In order to characterize the conserved sequence of CasR binding to the promoter of the *csm6*, we analyzed putative −35 (TTGAAG) and −10 (ATATGC) sequences upstream of the annotated translation start codon, based on transcription start site (Figure 5A). The results showed that the binding region of ribosomal protein was far from the translation start site. CasR-bound palindromic sequence alignment showed that there was a highly similar inverted repeat sequence in the region −331 to −301 of the *csm6* promoter (Figure 5A). We speculated that CasR might regulate the expression of *csm6* by directly binding to this region (−331 to −301). To test this speculation, we directly synthesized two 29 bp DNA probes, with or without IRs sequence, and performed the EMSA analysis. As shown in Figure 5B, CasR bound to *csm6p1* but failed to bind to *csm6p2*. These results indicate that the region (−331 to −301) where CasR bound to the *csm6* promoter was far from the transcription start site, and the sequence characteristics of this region are consistent with those of the CasR-binding motif.

### 3.5. CasR Negatively Regulates Expression of Gene csm6

**Figure 6:**
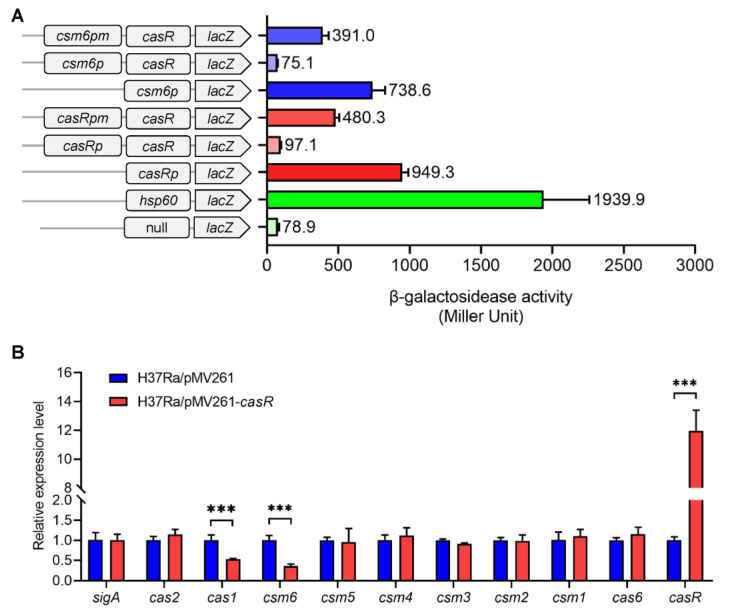
CasR negatively regulates the expression of *csm6*. (**A**) β-galactosidase activity assay. Schematic diagram of plasmids containing promoter-*lacZ* and promoter-CasR ORF-*lacZ*. All the plasmids were transformed into the *M. smegmatis*. The activity of β-galactosidase is expressed as Miller units. Data are expressed as the mean ± standard deviations of three independent replicates. (**B**) Relative expression levels of CRISPR-associated genes via qRT-PCR in the *casR*-overexpressing strain and pMV261 control strain. The relative expression levels of the genes were normalized using the *sigA* gene as internal control. Relative expression levels of genes were calculated using the 2^−ΔΔCt^ method. The differences in the relative expression levels between groups were analyzed via unpaired two-tailed Student’s *t*-test, using GraphPad Prism 8. Asterisks represent significant differences (***, *p* < 0.001) between two groups.

To investigate the regulation of *csm6* expression, we amplified a series of promoter fragments and cloned them into a promoter-less *lacZ* reporter plasmid and transformed these plasmids into *M. smegmatis*. The β-galactosidase activity analysis results showed that the strong promoter *hsp60* significantly promoted the expression of *lacZ,* and it exhibited higher enzymatic activity than the promoter-less *lacZ* plasmid, indicating that the reporter system worked well (Figure 6A). Furthermore, the *casRp* and *csm6p* promoters also significantly promoted the expression of *lacZ* (~949.3 and 738.6 Miller units). Notably, when the open reading frame (ORF) of *casR* was cloned behind the promoter, the enzymatic activity of *casRp*-*lacZ* and *csm6p*-*lacZ* was significantly reduced by about 10-fold (~97.1 and 75.1 Miller units). However, when the palindromic sequence motifs of *casRpm* and *csm6pm* were mutated and used as promoters, the expression level of *lacZ* in the mutant strain was drastically rescued when they had the *casR* with the mutated promoter (~480.3 and 391.0 Miller units) (Figure 6A). These results indicate that the transcription of *csm6* was suppressed by CasR.

The regulatory effect of CasR on *csm6* was further examined in the *M. tuberculosis* H_37_Ra strain, and the expression of CRISPR-associated genes was quantified with qRT-PCR. When the *casR* gene was overexpressed via the pMV261 vector with L5 regulatory promoter (Figure 6B), the expression of *casR* increased by more than 12-fold in the *casR*-overexpressing strain relative to the pMV261 strain, indicating that the *casR* gene was overexpressed successfully. In contrast, *csm6* expression significantly decreased by at least 2.5-fold in the *casR*-overexpressing strain. In addition, we detected the expression levels of other CRISPR-associated genes and found that the expression of *cas1* adjacent to the downstream of *csm6* was also downregulated (Figure 6B), but the expression of the distant *cas2* and upstream genes of *csm6* were not significantly changed, which was consistent with the EMSA results (Figure 6B).

Overall, our results suggested that CasR functioned as a transcriptional repressor, and that it negatively regulated the expressions of *csm6* and *cas1*, rather than other CRISPR-associated genes.

## 4. Discussion

The CRISPR-Cas system, as an adaptive immune system in many bacteria and archaea, can defend against the invasion of foreign nucleic acids, and this system has multiple functions such as gene expression regulation, genome evolution, and DNA repair [34,35]. The expression regulation of CRISPR-Cas system genes is essential for the functional exertion of this system. However, our knowledge of the expression regulation of CRISPR-Cas system genes is still limited. In this study, we found that transcription factor CasR specifically bound to the promoter of *csm6* and negatively regulated its expression.

The TetR family of transcription factors can regulate the expression of the genes related to diverse physiological functions, such as drug efflux pumps, catabolic pathway enzyme synthesis, antibiotic biosynthesis, osmotic stress, and the formation of pathogenic bacterial biofilms in bacteria [36,37,38,39,40]. In the current study, we identified and characterized a TetR family transcription factor CasR in *M. tuberculosis* H_37_Ra, and CasR was highly conserved in the strains of the *M. tuberculosis* complex. Our EMSA and ChIP results confirmed that CasR can bind its own promoter specifically, and it can also interact with the *csm6* promoter. It is noteworthy that the binding activity of CasR to its own promoter is much stronger than to the promoter of the *csm6* gene. In the EMSA experiment, the amount of CasR protein required to fully bind to its own promoter is about 1 μM, while 2 μM of the protein was needed to bind with the promoter of *csm6* (Figure 3B). In addition, our mutation analysis of the CasR-binding DNA motif showed that only one shifted band was detected using *casRp1* and *csm6p1* as probes, indicating that a single binding motif of the *casR* promoter is sufficient for CasR binding; however, this may not be true for the scenario of the *csm6* promoter (Figure 4D and Figure 5B). These results imply the presence of a cooperative binding effect, which has also been found for other TetR family regulators [27,36].

Our β-galactosidase analysis showed that CasR negatively regulated its own promoter expression and suppressed the expression of the CRISPR-associated gene *csm6* (Figure 6A). Our qRT-PCR results confirmed that the expression level of *csm6* was significantly reduced in the *casR*-overexpressing strain and that the expression of gene *cas1* was also reduced by about two-fold (Figure 6B). The binding site of CasR within its own promoter DNA is much closer to its coding sequence, compared with that of *csm6*. Furthermore, sequence alignment showed that palindromic sequences of *casR* and *csm6* promoters were highly conserved in *M. tuberculosis* H_37_Rv, *M. tuberculosis* H_37_Ra, and *M. bovis* BCG strains. Usually, the site recognized by RNA polymerase is near the −35 region, and our data showed that the palindromic sequence of CasR-binding *csm6* promoter was located upstream of the −35 region (Figure 5A). Therefore, CasR might prevent RNA polymerase from binding to the promoter region of *csm6*, thus resulting in the downregulation of gene expression. Although the ChIP-seq screening revealed that the region of CasR binding to the *csm6* gene was located in the coding region, we proved that its binding site was in the promoter region of *csm6* by both in vitro and in vivo experiments. This indicated that there are still uncertainties in the experimental results of ChIP-seq, and the results need to be verified by further experiments. We also analyzed whether there were CasR-binding palindrome sequences in the coding sequences of the *csm6* gene, and found only half a palindrome structure in the positions 994–1001. We hypothesized that CasR might also be able to weakly bind to the coding region of the *csm6* gene. The binding of CasR in the *csm6* coding region might act as a transcription roadblock and could also physically interfere with the transcription of *cas1*. The molecular mechanism by which CasR regulates the expression of CRISPR-Cas system genes can be further explored by analyzing the crystal structure of the CasR-*csm6* promoter complex.

The regulation of *cas* gene expression is particularly important for avoiding the over-immunity of the CRISPR-Cas system after bacteria clear the invaded nucleic acids. CnpB has been reported to regulate the expression of CRISPR-Cas system genes through nanoRNA, and CnpB upregulates the expression of all the *cas* genes globally [22], which was inconsistent with our results that CasR accurately regulated the expression level of *csm6* by binding to the promoter of the CRISPR system. In *E. coli*, the DNA-binding protein H-NS suppresses CRISPR-cas gene expression due to the fact that the H-NS binding sites are located near the *cas* operon promoters [41]. In *Salmonella enterica* Serovar Typhi, LRP is also a negative regulator for *cas* expression, with a fourfold inhibitory effect on the *casA* promoter [42]. Likewise, our findings indicate that CasR significantly inhibits the expression of the *csm6* gene. This accurate regulation was important for bacterial response to phage- or transposon- induced horizontal gene transfer, conjugation, and transduction of plasmids carrying resistance genes. However, how CasR receives upstream signals to regulate the expression of *cas* genes deserves to be further investigated. We hypothesized that after phage and plasmid removal, CasR could rapidly inhibit the expression of the *csm6* gene, in order to avoid the over-inhibition of bacterial growth. Based on the results of the overexpressing strain, we speculate that the mutation of *casR* might upregulate the gene expression of the CRISPR-Cas system. The regulable CRISPR-Cas system is necessary for the survival of bacteria, since it can save the cost of expressing a large number of cells and avoid excessive nuclease accumulation, thus enhancing bacterial immunity against foreign nucleic acid invasion.

In conclusion, this study confirmed the interaction of the TetR family CasR with its own promoter and the upstream DNA sequence of the CRISPR-Cas gene cluster identified the conserved DNA sequences recognized by CasR and preliminarily elucidated the regulatory effect of CasR on *cas* gene expression. Our findings provide valuable references for further studies on gene expression regulation in the CRISPR-Cas system.

## Data Availability

The data presented in this study are available on request from the corresponding author.

## Supplementary Materials

The following supporting information can be downloaded at: https://www.mdpi.com/article/10.3390/biom13020400/s1: Figure S1: SDS-PAGE of purified proteins; Figure S2: EMSA assays for the binding of Rv1255c to promoters; Figure S3: EMSA assays for the binding of Rv1473A to promoters; Figure S4: EMSA assays for the binding of CasR to promoters; Figure S5: The amino acid sequence alignment of CasR; Table S1: Bacterial strains and plasmids used in this study; Table S2: Primers used in this study.

## References

[1] Makarova K.S., Wolf Y.I., Iranzo J., Shmakov S.A., Alkhnbashi O.S., Brouns S.J.J., Charpentier E., Cheng D., Haft D.H., Horvath P. (2020). Evolutionary classification of CRISPR-Cas systems: A burst of class 2 and derived variants. Nat. Rev. Microbiol..

[2] Makarova K.S., Wolf Y.I., Koonin E.V. (2013). Comparative genomics of defense systems in archaea and bacteria. Nucleic Acids Res..

[3] Shmakov S., Smargon A., Scott D., Cox D., Pyzocha N., Yan W., Abudayyeh O.O., Gootenberg J.S., Makarova K.S., Wolf Y.I. (2017). Diversity and evolution of class 2 CRISPR-Cas systems. Nat. Rev. Microbiol..

[4] Weinberger A.D., Gilmore M.S. (2015). A CRISPR view of cleavage. Cell.

[5] Makarova K.S., Wolf Y.I., Alkhnbashi O.S., Costa F., Shah S.A., Saunders S.J., Barrangou R., Brouns S.J., Charpentier E., Haft D.H. (2015). An updated evolutionary classification of CRISPR-Cas systems. Nat. Rev. Microbiol..

[6] Nussenzweig P.M., Marraffini L.A. (2020). Molecular Mechanisms of CRISPR-Cas Immunity in Bacteria. Annu. Rev. Genet..

[7] Strotskaya A., Savitskaya E., Metlitskaya A., Morozova N., Datsenko K.A., Semenova E., Severinov K. (2017). The action of *Escherichia coli* CRISPR-Cas system on lytic bacteriophages with different lifestyles and development strategies. Nucleic Acids Res..

[8] Brouns S.J., Jore M.M., Lundgren M., Westra E.R., Slijkhuis R.J., Snijders A.P., Dickman M.J., Makarova K.S., Koonin E.V., van der Oost J. (2008). Small CRISPR RNAs guide antiviral defense in prokaryotes. Science.

[9] Guo T.W., Bartesaghi A., Yang H., Falconieri V., Rao P., Merk A., Eng E.T., Raczkowski A.M., Fox T., Earl L.A. (2017). Cryo-EM structures reveal mechanism and inhibition of DNA targeting by a CRISPR-Cas surveillance complex. Cell.

[10] Sternberg S.H., Richter H., Charpentier E., Qimron U. (2016). Adaptation in CRISPR-Cas systems. Mol. Cell.

[11] Westra E.R., Swarts D.C., Staals R.H., Jore M.M., Brouns S.J., van der Oost J. (2012). The CRISPRs, they are a-changin’: How prokaryotes generate adaptive immunity. Annu. Rev. Genet..

[12] Freidlin P.J., Nissan I., Luria A., Goldblatt D., Schaffer L., Kaidar-Shwartz H., Chemtob D., Dveyrin Z., Head S.R., Rorman E. (2017). Structure and variation of CRISPR and CRISPR-flanking regions in deleted-direct repeat region *Mycobacterium tuberculosis* complex strains. BMC Genom..

[13] Pourcel C., Touchon M., Villeriot N., Vernadet J.P., Couvin D., Toffano-Nioche C., Vergnaud G. (2020). CRISPRCasdb a successor of CRISPRdb containing CRISPR arrays and cas genes from complete genome sequences, and tools to download and query lists of repeats and spacers. Nucleic Acids Res..

[14] Tamulaitis G., Venclovas C., Siksnys V. (2017). Type III CRISPR-Cas immunity: Major differences brushed aside. Trends Microbiol..

[15] Supply P., Marceau M., Mangenot S., Roche D., Rouanet C., Khanna V., Majlessi L., Criscuolo A., Tap J., Pawlik A. (2013). Genomic analysis of smooth tubercle bacilli provides insights into ancestry and pathoadaptation of *Mycobacterium tuberculosis*. Nat. Genet..

[16] Wei W., Zhang S., Fleming J., Chen Y., Li Z., Fan S., Liu Y., Wang W., Wang T., Liu Y. (2019). *Mycobacterium tuberculosis* type III-A CRISPR/Cas system crRNA and its maturation have atypical features. FASEB J..

[17] Gruschow S., Athukoralage J.S., Graham S., Hoogeboom T., White M.F. (2019). Cyclic oligoadenylate signalling mediates *Mycobacterium tuberculosis* CRISPR defence. Nucleic Acids Res..

[18] Jiang W., Samai P., Marraffini L.A. (2016). Degradation of phage transcripts by CRISPR-associated RNases enables type III CRISPR-Cas immunity. Cell.

[19] Athukoralage J.S., Graham S., Grüschow S., Rouillon C., White M.F. (2019). A type III CRISPR ancillary ribonuclease degrades its cyclic oligoadenylate activator. J. Mol. Biol..

[20] Athukoralage J.S., Rouillon C., Graham S., Gruschow S., White M.F. (2018). Ring nucleases deactivate type III CRISPR ribonucleases by degrading cyclic oligoadenylate. Nature.

[21] Richter C., Chang J.T., Fineran P.C. (2012). Function and regulation of clustered regularly interspaced short palindromic repeats (CRISPR)/CRISPR associated (Cas) systems. Viruses.

[22] Zhang Y., Yang J., Bai G. (2018). Regulation of the CRISPR-associated genes by Rv2837c (CnpB) via an orn-like activity in tuberculosis complex mycobacteria. J. Bacteriol..

[23] Wei W., Qiao J., Jiang X., Cai L., Hu X., He J., Chen M., Yang M., Cui T. (2022). Dehydroquinate synthase directly binds to streptomycin and regulates susceptibility of *Mycobacterium bovis* to streptomycin in a non-canonical mode. Front. Microbiol..

[24] Yang M., Gao C., Cui T., An J., He Z.G. (2012). A TetR-like regulator broadly affects the expressions of diverse genes in *Mycobacterium smegmatis*. Nucleic Acids Res..

[25] Yang M., Gao C.H., Hu J., Zhao L., Huang Q., He Z.G. (2015). InbR, a TetR family regulator, binds with isoniazid and influences multidrug resistance in *Mycobacterium bovis* BCG. Sci. Rep..

[26] Zhu C., Liu Y., Hu L., Yang M., He Z.G. (2018). Molecular mechanism of the synergistic activity of ethambutol and isoniazid against *Mycobacterium tuberculosis*. J. Biol. Chem..

[27] Gao C.H., Yang M., He Z.G. (2012). Characterization of a novel ArsR-like regulator encoded by Rv2034 in *Mycobacterium tuberculosis*. PLoS ONE.

[28] Livak K.J., Schmittgen T.D. (2001). Analysis of relative gene expression data using real-time quantitative PCR and the 2(-Delta Delta C(T)) Method. Methods.

[29] Cortes T., Schubert O.T., Rose G., Arnvig K.B., Comas I., Aebersold R., Young D.B. (2013). Genome-wide mapping of transcriptional start sites defines an extensive leaderless transcriptome in *Mycobacterium tuberculosis*. Cell Rep..

[30] Jaini S., Lyubetskaya A., Gomes A., Peterson M., Tae Park S., Raman S., Schoolnik G., Galagan J. (2014). Transcription factor binding site mapping using ChIP-Seq. Microbiol. Spectr..

[31] Galagan J.E., Minch K., Peterson M., Lyubetskaya A., Azizi E., Sweet L., Gomes A., Rustad T., Dolganov G., Glotova I. (2013). The *Mycobacterium tuberculosis* regulatory network and hypoxia. Nature.

[32] Gao C.H., Yang M., He Z.G. (2011). An ArsR-like transcriptional factor recognizes a conserved sequence motif and positively regulates the expression of *phoP* in mycobacteria. Biochem. Biophys. Res. Commun..

[33] Sherman D.R., Voskuil M., Schnappinger D., Liao R., Harrell M.I., Schoolnik G.K. (2001). Regulation of the *Mycobacterium tuberculosis* hypoxic response gene encoding alpha -crystallin. Proc. Natl. Acad. Sci. USA.

[34] Mogila I., Kazlauskiene M., Valinskyte S., Tamulaitiene G., Tamulaitis G., Siksnys V. (2019). Genetic dissection of the type III-A CRISPR-Cas system Csm complex reveals roles of individual subunits. Cell Rep..

[35] Westra E.R., Buckling A., Fineran P.C. (2014). CRISPR-Cas systems: Beyond adaptive immunity. Nat. Rev. Microbiol..

[36] Ramos J.L., Martínez-Bueno M., Molina-Henares A.J., Terán W., Watanabe K., Zhang X., Gallegos M.T., Brennan R., Tobes R. (2005). The TetR family of transcriptional repressors. Microbiol. Mol. Biol. Rev..

[37] Balhana R.J., Singla A., Sikder M.H., Withers M., Kendall S.L. (2015). Global analyses of TetR family transcriptional regulators in mycobacteria indicates conservation across species and diversity in regulated functions. BMC Genom..

[38] Miotto P., Sorrentino R., De Giorgi S., Provvedi R., Cirillo D.M., Manganelli R. (2022). Transcriptional regulation and drug resistance in *Mycobacterium tuberculosis*. Front. Cell. Infect. Microbiol..

[39] Wang K., Sybers D., Maklad H.R., Lemmens L., Lewyllie C., Zhou X., Schult F., Bräsen C., Siebers B., Valegård K. (2019). A TetR-family transcription factor regulates fatty acid metabolism in the archaeal model organism *Sulfolobus acidocaldarius*. Nat. Commun..

[40] Carette X., Blondiaux N., Willery E., Hoos S., Lecat-Guillet N., Lens Z., Wohlkönig A., Wintjens R., Soror S.H., Frénois F. (2012). Structural activation of the transcriptional repressor EthR from *Mycobacterium tuberculosis* by single amino acid change mimicking natural and synthetic ligands. Nucleic Acids Res..

[41] Pul U., Wurm R., Arslan Z., Geissen R., Hofmann N., Wagner R. (2010). Identification and characterization of *E. coli* CRISPR-cas promoters and their silencing by H-NS. Mol. Microbiol..

[42] Medina-Aparicio L., Rebollar-Flores J.E., Gallego-Hernández A.L., Vázquez A., Olvera L., Gutiérrez-Ríos R.M., Calva E., Hernández-Lucas I. (2011). The CRISPR/Cas immune system is an operon regulated by LeuO, H-NS, and leucine-responsive regulatory protein in Salmonella enterica serovar Typhi. J. Bacteriol..

