# Supplementary material for: Regulation of CRISPR-Associated Genes by Rv1776c (CasR) in Mycobacterium tuberculosis
Source: Biomolecules. 2023 Feb 20;13(2):400. doi: 10.3390/biom13020400 (PMC9953421; doi:10.3390/biom13020400)
Supplement: Supplementary file 1 [file biomolecules-13-00400-s001.zip › biomolecules-2097224-supplementary.pdf]

Supplementary Materials

Supplementary Table S1. Bacterial strains and plasmids used in this study.

Strains and plasmids	Relevant genotype or features	Source
**Strain**		
E.coli BL21	Host for overexpression	Novagen
M. smegmatis MC^2 155	*M. smegmatis* MC^2 155 wild type	ATCC
Ms/pMV261-null	$mc^2$155 with pMV261-*lacZ*, negative control	This study
Ms/pMV261-*hsp60*	$mc^2$155 with pMV261-*hsp60*-*lacZ*, positive	This study
Ms/pMV261-*casRp*	$mc^2$155 with pMV261-*casRp*-*lacZ*	This study
Ms/pMV261-*casRp*-*casR*	$mc^2$155 with pMV261-*casRp*-*casR*-*lacZ*	This study
Ms/pMV261-*casRpm*-*casR*	$mc^2$155 with pMV261-*casRpm*-*casR*-*lacZ*	This study
Ms/pMV261-*csm6p*	$mc^2$155 with pMV261-*csm6p*-*lacZ*	This study
Ms/pMV261-*csm6p*-*casR*	$mc^2$155 with pMV261-*csm6p*-*casR*-*lacZ*	This study
Ms/pMV261-*csm6pm*-*casR*	$mc^2$155 with pMV261-*csm6pm*-*casR*-*lacZ*	This study
M. tuberculosis H_{37}Ra	*M. tuberculosis* H_{37}Ra wild type	ATCC
H_{37}Ra /pMV261	H_{37}Ra with pMV261	This study
H_{37}Ra /pMV261-*casR*	*casR*-overexpressing strain	This study
**Plasmid**		
pET28a	Kan^r, *lacZ* operon, T7 promotor, N-termial His-Tag	Novagen
pET28a-*Rv0324*	*Rv0324* inserted in *Eco*RI-*Xba*I of pET28a	This study
pET28a-*Rv1255c*	*Rv1255c* inserted in *Eco*RI-*Xba*I of pET28a	This study
pET28a-*Rv1473A*	*Rv1473A* inserted in *Eco*RI-*Xba*I of pET28a	This study
pET28a-*casR*	*casR* inserted in *Eco*RI-*Xba*I of pET28a	This study
pET28a-*Rv0047c*	*Rv0047c* inserted in *Eco*RI-*Xba*I of pET28a	This study
pMV261	Kan^r, pAL5000 replicon	This study
pMV261-*casR*	*casR* inserted in *Eco*RI-*Xba*I of pMV261	This study

Supplementary Table S2. Primers used in this study.

Name	Sequence (5′-3′)	Note
casR-F	TATAGAATTCTAGTGCCGGGTAACGATTGGAT	Clone and expression
casR-R	TATATATCTAGATCAGCTTTGCTCGGCAAACG	Clone and expression
Rv0324-F	ATAAGAATTCGAATGGCTGGACAGTCCGATCG	Clone and expression
Rv0324-R	ATAATCTAGAAATCAATCCCCATGCCCGACCG	Clone and expression
Rv1255c-F	ATATGAATTCGCATGGCGGGTACCGACTGGCT	Clone and expression
Rv1255c-R	ATATGCTCTAGATCACTCGGGTCCAGGGTGAC	Clone and expression
Rv1473A-F	GCGCGAATTCGCATGCGGAAGTCAAAGAAGAC	Clone and expression
Rv1473A-R	ATATATTCTAGATCAACGCGGCCGGGAACGGC	Clone and expression
Rv0047c-F	ATAAGAATTCAAATGCTGGAGCTCGCCATCCT	Clone and expression
Rv0047c-R	ATAATCTAGAAATTACGTCTGTTCGGCGGGGT	Clone and expression
casR-pMV261-F	TGCTGAATTCGTGCCGGGTAACGATTGGAT	Clone to pMV261
casR-pMV261-R	AGTCTCTAGATCAGCTTTGCTCGGCAAACG	Clone to pMV261
casRp-F	CGTATAGGTCATCGTGGCGG	EMSA and ChIP, PCR
casRp-R1	TGAGCCCGTAGCGCGTAATG	EMSA, PCR
casRp-R2	TGGTCAGGCGCATTTCCATC	ChIP, PCR
csm6p-F	CCGAGACAGCCGCGTCCGTG	EMSA and ChIP, PCR
csm6p-R1	CTATGCGAACGTCGGTCTCA	EMSA, PCR
csm6p-R2	GACAACTATTGGTCGATAGT	ChIP, PCR
cas6p-F	GGTGCGCGCCGACCGGCTCG	EMSA and ChIP, PCR
cas6p-R1	TCAAGCCTGATTCCGCCGCA	EMSA, PCR
cas6p-R2	GGGATGGCTTCCTAACGAGC	ChIP, PCR
Rv0324p-F	CGTCAGCTCGTTGAGCGGGA	EMSA, PCR
Rv0324p-R	GCAAGTCCAGGATTTGCAAT	EMSA, PCR
Rv1255cp-F	CAGAGGGCATCCCCGACGCG	EMSA, PCR
Rv1255cp-R	ACGCCGGGTCACGCTGCGTA	EMSA, PCR
Rv1473Ap-F	CGGTCCGCAGCTCGAGCAGC	EMSA, PCR
Rv1473Ap-R	GGCCGGTGGTGGCCGCCAGG	EMSA, PCR
groEL1p-F	ATCGTGGCGAAGGTGAACAT	EMSA, PCR
groEL1p-R	CGCGCGGCCCCAGCGTCACC	EMSA, PCR
csm6 RT-F	TCAAGTGAGCAATCTGATCCG	qRT-PCR
csm6 RT-R	ATGTACTCAGCGACCTTGTTC	qRT-PCR
csm5 RT-F	CGAAAGGTTTGAGCGGAAGG	qRT-PCR
csm5 RT-R	TTCATGTCCATCTTCTGGCA	qRT-PCR

Supplementary Table S2. *Cont*.

Name	Sequence (5′-3′)	Note
csm4 RT-F	TCCGGTTCGAGCTGGACGCG	qRT-PCR
csm4 RT-R	GTGCTTCTGACTCGGTAAGG	qRT-PCR
csm3 RT-F	TTGCTGTCCCGCCAATAC	qRT-PCR
csm3 RT-R	TGTTGGTGAGCTTCGTGTC	qRT-PCR
csm2 RT-F	ACGACTATGTGAAACAGGCC	qRT-PCR
csm2 RT-R	GGTTGTGGTCAGCTCGAAG	qRT-PCR
csm1 RT-F	GGCAAGTAACCGCGCCCTCC	qRT-PCR
csm1 RT-R	ACGCTGCATTCCCGGTCACC	qRT-PCR
cas6 RT-F	ACGAGGCGCGGCAGCAGATC	qRT-PCR
cas6 RT-R	CGGGCCGCGCGTAGAAAATG	qRT-PCR
cas2 RT-F	TCAATCTCCCGCTCAAAGTG	qRT-PCR
cas2 RT-R	GGTTGTCGCTGATGTCGTAT	qRT-PCR
cas1 RT-F	TTCAGCAAGAACTCCGACAC	qRT-PCR
cas1 RT-R	CTGAAAGGTGTATCGGTGAGG	qRT-PCR
sigA RT-F	GGAGAAGTTCGACTACACCAAG	qRT-PCR
sigA RT-R	GTTGATCACCTCGACCATGT	qRT-PCR

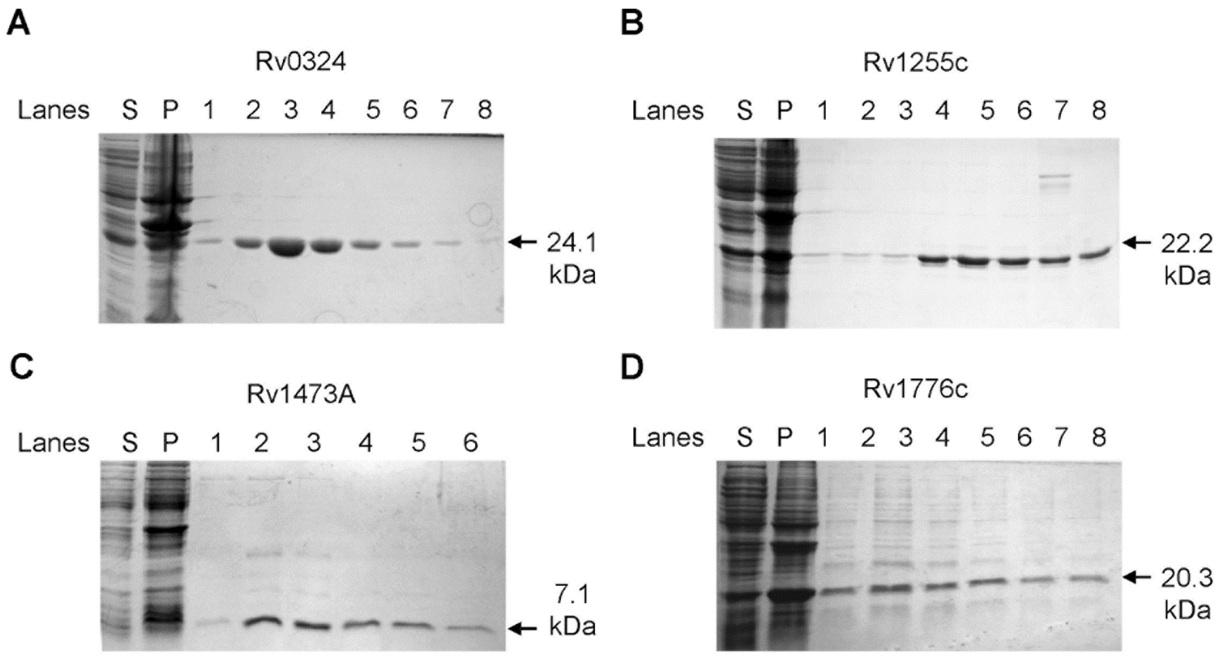

Supplementary Figure S1. SDS-PAGE of purified proteins. The purified proteins **(A)** Rv0324, **(B)** Rv1255c, **(C)** Rv1473A, and **(D)** Rv1776c were visualized by SDS-PAGE analysis. S represents supernatant, P represents pellet, and Lanes 1–8 represent purified samples.

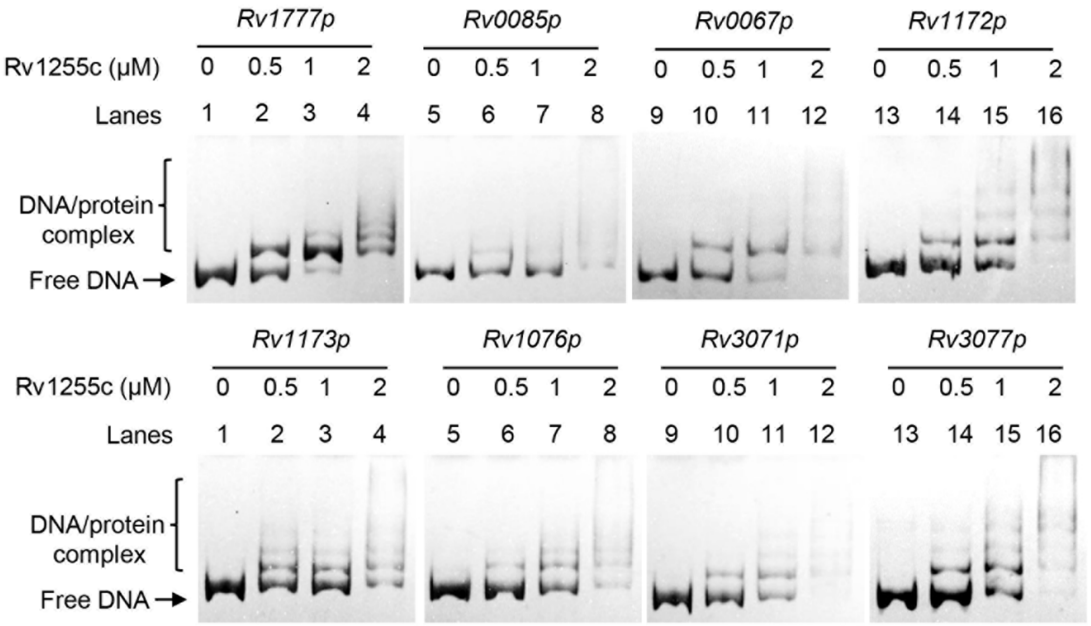

Supplementary Figure S2. EMSA assays for the binding of Rv1255c to promoters. Promoter fragments were co-incubated with various amount of Rv1255c protein as indicated. Rv1255c bound to all test promoter fragments and form stable DNA/protein complexes.

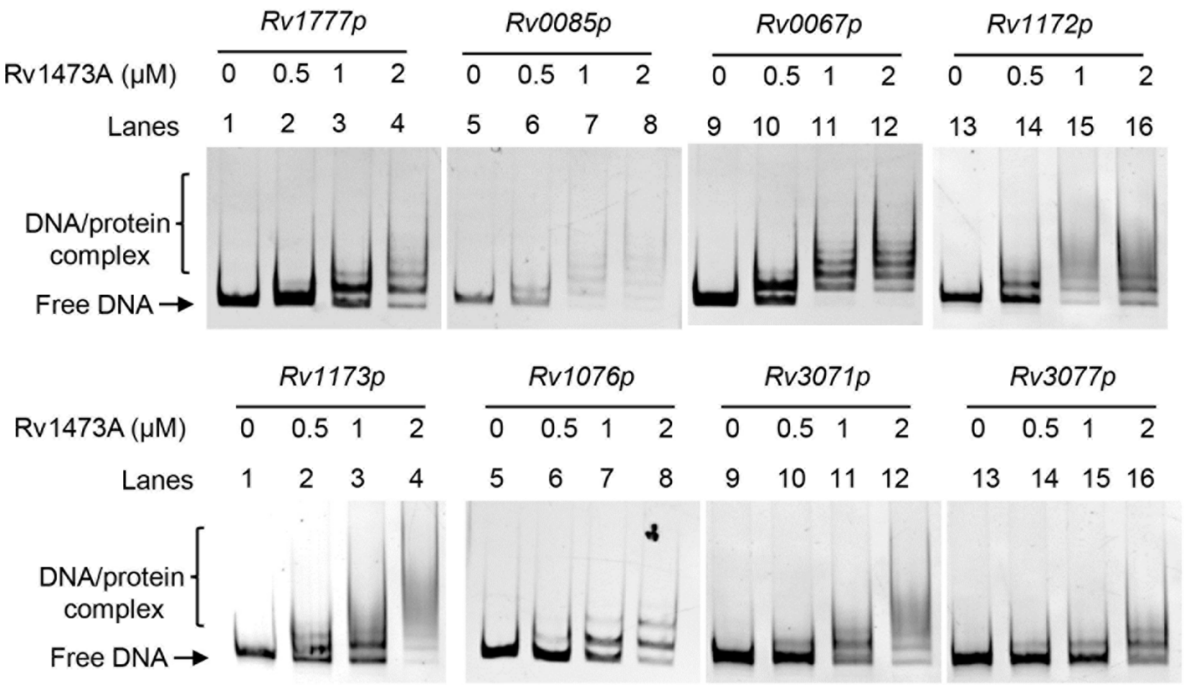

Supplementary Figure S3. EMSA assays for the binding of Rv1473A to promoters. Promoter fragments were co-incubated with various amount of Rv1473A protein as indicated. Rv1473A bound to all test promoter fragments and form stable DNA/protein complexes.

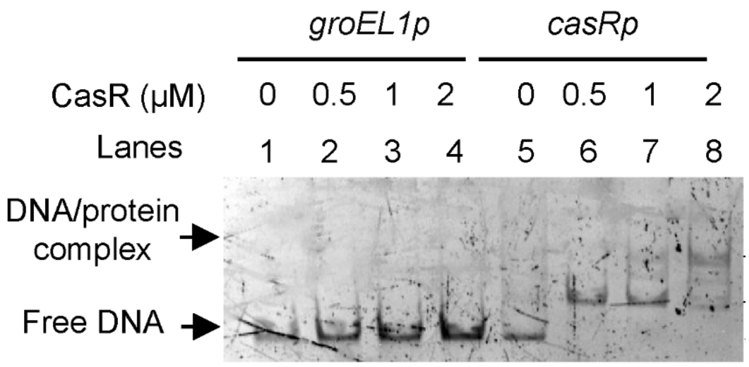

Supplementary Figure S4. EMSA assays for the binding of CasR to promoters. *casRp* or *groEL1p* were co-incubated with various amount of CasR protein as indicated. CasR bound to *casRp*, but not to *groEL1p*, and form stable DNA/protein complexes.

```
Rv1776c         MPGNDWIVGGNRRTIAAERIYAAATDLITRYGLNALDIDKLAREVHCSRATIYRR 55
MRA_1792        MPGNDWIVGGNRRTIAAERIYAAATDLITRYGLNALDIDKLAREVHCSRATIYRR 55
BCG_1809c       MPGNDWIVGGNRRTIAAERIYAAATDLITRYGLNALDIDKLAREVHCSRATIYRR 55
MSMEG_0676      --MADWLLGGDRRTAAAERIYAAATELVLRDGLDAFDIDTLAARVHCSRATVYRY 53
                    **::**:*** **********:*: * **:*:***.** .*******:** 

Rv1776c         AGGKAQIRDVVLTRAAARIADGVRSDVETLRGRERVVAAILLSLQRIRSDPLGKL 110
MRA_1792        AGGKAQIRDVVLTRAAARIADGVRSDVETLRGRERVVAAILLSLQRIRSDPLGKL 110
BCG_1809c       AGGKAQIRDVVLTRAAARIADGVRSDVETLRGRERVVAAILLSLQRIRSDPLGKL 110
MSMEG_0676      AGGKAQIRDAVLLRLAASIVDSVRQAVGHLSGRERVVRAVTVALEHIRSDPIRQM 108
                *********.** * ** *.*.**. *  * ****** *: ::*::*****: ::

Rv1776c         MFGSIHGGAGELAWLTESPLLADFATELTGIAGGDPQGAKWVVRVVLSLMYWPAE 165
MRA_1792        MFGSIHGGAGELAWLTESPLLADFATELTGIAGGDPQGAKWVVRVVLSLMYWPAE 165
BCG_1809c       MFGSIHGGAGELAWLTESPLLADFATELTGIAGGDPQGAKWVVRVVLSLMYWPAE 165
MSMEG_0676      MVGLG--AGRDLSELPASPVLADLAAELTGVT-DDPQAAQWIVRVVMSLAVWPIG 150
                *.*    .. :*: *  **:***:*:****:: .***.*:*:****:**  **  

Rv1776c         NDEAERRLVEKYVAPAFAEQS 186
MRA_1792        NDEAERRLVEKYVAPAFAEQS 186
BCG_1809c       NDEAERRLVEKYVAPAFAEQS 186
MSMEG_0676      DAAAEAGLVERFVAPAFE--- 178
                :  **  ***::*** **   
```

Supplementary Figure S5. The amino acid sequence alignment of CasR. Multiple amino acid sequence alignment of CasR protein from *M. tuberculosis* H37Rv (Rv1776c), *M. tuberculosis* H37Ra (MRA_1792), *M. bovis* BCG (BCG_1809c), and *M. smegmatis* (MSMEG_0676) using ClustalOmega.
